# In a rodent model of autism, probiotics decrease gut leakiness in relation to gene expression of GABA receptors: Emphasize how crucial the gut–brain axis

**DOI:** 10.1515/tnsci-2022-0354

**Published:** 2024-10-03

**Authors:** Rawan M. Bin-Khattaf, Abeer M. Al-Dbass, Mona Alonazi, Ramesa Shafi Bhat, Sooad Al-Daihan, Afaf K. El-Ansary

**Affiliations:** Biochemistry Department, Science College, King Saud University, P.O. Box 22452, Riyadh, 11495, Saudi Arabia; Autism Center, Lotus Holistic Medical Center, Abu Dhabi, 110281, United Arab Emirates

**Keywords:** autism spectrum disorders, propionic acid, probiotics, zonulin, occludin, GABA receptors, oxidative stress

## Abstract

**Objective:**

Rodent models may help investigations on the possible link between autism spectrum disorder and increased permeability of the gastrointestinal (GI) tract since autistic patients frequently manifested GI troubles as comorbidities.

**Methods:**

Forty young male western Albino rats, weighing approximately 60–70 g and aged 3–4 weeks, were used. In each of the six experimental groups, eight animals were treated as follows. The mice in the control group (I) received phosphate-buffered saline orally. For 3 days, the animals in the propionic acid (PPA)-treated groups (II and III) were given an oral neurotoxic dose of PPA (250 mg/kg body weight each day). Group II was euthanized after 3 days; however, Group III was left alive to be euthanized alongside the other groups. The animals were kept at 22 ± 1°C and allowed to access water and normal food as needed. Identical dosages of PPA were given to the rats in the three treatment groups (IV, V, and VI), and for 3 weeks, they were given the following treatments: 0.2 g/kg body weight of pure *Bifidobacterium infantis*, a probiotic mixture of PROTEXIN®, Somerset, UK and pure *Lactobacillus bulgaricus*, respectively. The six groups underwent measurements of serum zonulin and occludin as variables associated with leaky gut, glutathione, malondialdehyde, and catalase as oxidative stress-related variables, with gamma-aminobutyric acid (GABA) receptor gene expression.

**Results:**

This study demonstrated the potential effects of pure or mixed probiotics in lowering zonulin and occludin as markers of increased intestinal permeability, enhancing GABA receptor expression, and reducing oxidative stress as neurotoxic effects of PPA.

**Conclusions:**

This study demonstrates that various probiotics protect gut barrier function and could be used to alleviate increased intestinal permeability caused by oxidative stress and impaired GABA signaling as a result of PPA neurotoxicity, addressing the clinical implications of probiotic supplements.

## Abbreviations

ASD: Autism spectrum disorder
BBB: Blood–brain barrier
CFU: Colony-forming unit
CV%: Coefficient of variability
DTNB: 5,5-Diothio-bis-2-(nitrobenzoic acid)
GABA: γ-Amino-butyric acid
GABARA: γ-Amino-butyric acid A receptors
GABARB: γ-Amino-butyric acid type B receptors
GABARC: γ-Amino-butyric acid C receptors
GI: Gastrointestinal
GSH: Glutathione
ENS: Enteric nervous system
LGG: *Lactobacillus rhamnosus* GG
LPS: Lipopolysaccharides
MDA: Malondialdehyde
MGBA: Microbial–gut–brain axis
PPA: Propionic acid
SPSS: Statistical Program for Social Science
TJ: Tight junction
TLR: Toll-like receptors
ZO-1: Zonula occludens-1

## 1 Introduction

The gastrointestinal (GI) morbidity has been connected to autism spectrum disorder (ASD). Nearly half of children with ASD have at least one GI symptom as comorbidity [1,2]. Individuals with ASD are more likely than their neurotypical peers to have GI symptoms, with diarrhea and constipation being the most commonly reported GI problems [3,4,5]. A link between the severity of GI symptoms and the severity of ASD has been reported [6,7,8]. These findings suggest that the GI environment may have a role in the development of ASD.

According to reports, autistic patients had higher amounts of *Proteobacteria*, *Lactobacillus*, *Bacteroides*, *Desulfovibrio*, and *Clostridium*, while *Bifidobacterium*, *Blautia*, *Dialister*, *Prevotella*, and *Veillonella* were consistently lower [9]. *Proteobacteria*, which are plentiful in the guts of autistic people, are linked to host inflammation [10]. According to animal research*, Proteobacteria* produce lipopolysaccharide (LPS), which can lower the level of glutathione (GSH), an antioxidant, in the brain [11,12]. Inadequate synthesis of protective bacterial metabolites and increase of bacterial toxic metabolites may negatively impact gut–brain communication, supporting GI-related gut–brain diseases, among which are ASD.

The “leaky gut” theory and the gut–brain axis propose a route for these altered metabolites to enter the circulation and have an immediate effect on neurodevelopment [13]. A key role in the pathogenesis of ASD has been recently attributed to the gut microbiota, as it influences central nervous system development and neuropsychological and GI homeostasis through the microbiota–gut–brain axis [14,15].

The zonula occludens (ZO-1) toxin is an enterotoxin produced by gut epithelial cells in response to dietary or microbial stimuli. It has a strong influence on tight junction (TJ) capacity and intestinal barrier function. Notably, the disruption of the intestinal barrier in response to overgrowth of pathogenic bacteria allows pro-inflammatory cytokines to cross from the gut to the blood. Zonulin inhibits the production of intestinal TJ proteins, causes T-cell-mediated mucosal inflammation, and regulates immune cell transmigration from the gut to the blood [16]. Zonalin has been shown to cause the breakdown of TJs resulting in leaky gut [16] Serum zonulin levels have been associated with increased gut leakiness, which may have an influence on immune, hormonal, and neurological pathways. As a result, people with leaky gut may develop neurological disorders [17]. Moreover, occludin is a TJ protein that has been linked to epithelial permeability [18]. Occludin helps to maintain the stability and integrity of TJs, and this regulates and limits the paracellular transport pathway [19].

In ASD and anxiety disorders, there is a glutamatergic/GABAergic imbalance, with increased glutamatergic neurotransmission as an excitatory neurotransmitter and decreased gamma-aminobutyric acid (GABA) as an inhibitory neurotransmitter [20,21]. Glutamate increase causes excitotoxicity, which causes neuronal injury, cell death, and surviving neuron malfunction; however, delayed disruption of excitatory glutamate circuits causes deficiencies in cognitive and motor functions. GABA controls excitatory pathways in the brain, and the loss of GABA-producing cells after damage alters the balance of excitation and inhibition, leading to further cell destruction and apoptosis [22].

In people with autism, there is an increase in the abundance of *Clostridium*, the primary producer of propionate. Endotoxins and propionate produced by *Clostridium* may be linked to the severity of the clinical presentation of ASD. Interestingly, Strati et al. [23] reported that *Candida* was two times more common in patients with ASD than in the general population. High quantities of *Candida albicans* produce ammonia, which in the GI tract interacts with propionic acid (PPA) to make beta-alanine, which shares chemical similarities with GABA. The blood–brain barrier (BBB) can be crossed by beta-alanine, which also acts as a partial GABA antagonist by partially inhibiting GABA receptors. Although a large amount of research has shown GABA as a mediator within the enteric nervous system (ENS) modulating GI function, the full significance of GABAergic signaling in the gut remains unknown. GABA effects in the GI tract are dependent on the activation of ionotropic GABA_A_ and GABA_C_ receptors as well as metabotropic GABA_B_ receptors, potentially resulting in a notable control of both excitatory and inhibitory signaling in the ENS.

It is commonly known that PPA, a metabolite often produced by enlarged bacteria like clostridial and others, can be effectively employed to cause chronic autistic characteristics in mice [24].

This information tempers our interest to study the potential ameliorative effects of selected probiotics on gut leakiness and oxidative stress in a PPA-induced rodent model of ASD. The PPA model was chosen because it exhibited various behavioral and neuro-inflammatory alterations associated with ASD, is a byproduct of enteric bacteria, and has the ability to cross the gut–blood and gut–brain barriers [24,25].

While serum zonulin and occludin were measured as leaky gut biomarkers, GSH, catalase, and malondialdehyde (MDA) were used as measure of oxidative stress. Our study was extended to further demonstrate the cause-and-effect relationships between gut leakiness and gene expression of brain GABA receptors previously studied in the same rat model.

## 2 Materials and methods

### 2.1 Animals

In this investigation, 40 young male western Albino rats, weighing around 60–70 g and aged 3–4 weeks, were acquired from the animal laboratory and experimental surgery. Eight rats were included in each of the six experimental groups that were randomly assigned to the animals. For the mice in the control group (I), phosphate-buffered saline was given orally. For 3 days, the animals in the PPA-treated groups (II and III) received an oral neurotoxic dose of PPA (250 mg/kg body weight/day). Group II was euthanized after 3 days, while Group III was kept alive to be euthanized with other groups [16]. The rats in the three probiotics-treated groups (IV, V, and VI) were given the identical dosages of PPA for 3 days, after which they were given 0.2 g/kg body weight of ProtexinR, a probiotic, and the beneficial bacteria *Bifidobacterium infantis* and *Lactobacillus bulgaricus*, respectively. The selection of ProtexinR involves strains of microbes that exhibit the most favorable effects, safety, and the benefit-to-risk ratio associated with the usage of a certain probiotic strain. With a concentration of 1 billion CFU per gram, ProtexinR (Somerset, UK) is a mixture of various beneficial bacteria, including *Lactobacillus acidophilus*, *Lactobacillus bulgaricus*, *Lactobacillus casei*, *Lactobacillus rhamnosus* GG (LGG), and *Streptococcus thermophiles*. The rats were placed at 22 ± 1°C with *ad libitum* access to water and standard chow.

### 2.2 Preparation of brain tissue homogenates

Deeply ketamine/xylazine-anaesthetized animals were killed at the end of the feeding trial. The brain tissues were extracted from the six groups of rats and dissected into small pieces before being homogenized in bi-distilled water (1:10, w/v) and kept at 30°C until further use.

### 2.3 Biochemical assays

#### 2.3.1 Measurement of serum zonulin and occludin

Serum zonulin and occludin levels were measured in all groups using MyBioSource ELISA kits according to the manufacturer’s instructions (Catalog numbers: MBS2606662 and MBS725124, respectively). All measurements were taken in triplicate, and the average of the three readings was calculated. Quality control tests were carried out to assess experimental reproducibility using the inter- and intra-assay coefficients of variability (%CV).

### 2.4 Measurement of serum oxidative stress-related variables

#### 2.4.1 Lipid peroxidation concentration measurement

Lipid oxidation was measured using the Potter et al.’s approach, which looks for the production of thiobarbituric acid-reactive compounds [26].

#### 2.4.2 GSH assay

The GSH concentration was ascertained using 5,5′-dithiobis 2-nitrobenzoic acid in combination with sulfhydryl compounds to yield a reasonably persistent yellow hue, in accordance with the methodology outlined by Beutler et al. [27].

#### 2.4.3 Catalase activity assay

Catalase activity was examined using the Chance and Maehly technique [28], which involved monitoring the enzyme rate of dissociation of hydrogen peroxide per minute.

### 2.5 Gene expression

The gene expression of GABA in brain tissue was determined according to the method of our previously published work [29].

### 2.6 Statistical analysis

The Statistical Program for Social Sciences (SPSS) (SPSS Inc., Chicago, IL, USA) was used in all analyses. The data were presented as a mean ± standard deviations. All statistical comparisons were made using the Student’s *t*-test. *P* values less than 0.05 were considered significant. The correlation between the measured parameters was determined using multiple regression analysis with the SPSS program. In this study, adjusted *R* ^2^ refers to the proportion or percentage of variance in the dependent variable that can be explained by the variance in the independent variables, also known as predictors. An *R* ^2^ of 1.00 means that the independent factors account for all variations in the dependent variable. In contrast, an adjusted *R* ^2^ of 0.0 shows that the dependent variable is unaffected by the independent factors.


**Ethical approval:** The research related to animals’ use has been complied with all the relevant national regulations and institutional policies for the care and use of animals. The experiment protocol was in accordance with the ethical standards of the ethics committee responsible for animal experimentation at King Saud University, Riyadh, and was approved according to the Helsinki Declaration of 1975, as revised in 2008 (http://www.wma.net/en/20activities/10ethics/10helsinki/, accessed on 18 June 2022). (IRB NO.: KSU-SE-19-131).

## 3 Results

Table 1 shows the effects of *Lactobacillus* and *Bifidobacteria*, either alone or in combination, on serum zonulin and occludin, biomarkers of intestinal permeability caused by neurotoxic dosages of PPA provided in an experiment. Both probiotics demonstrated anti-gut leakiness effects, as seen by significantly reduced blood zonulin levels, even though the PPA-induced ASD animal model had significantly higher levels of both variables.

**Table 1:** Serum zonulin and occludin levels in the control, PPA-induced ASD model, and probiotics-treated groups

Group	Zonulin	Occludin
Control	3.157 ± 0.634	4.015 ± 0.165
PPA	8.701 ± 0.843*a	4.798 ± 1.539
PPA+	7.466 ± 1.043*a	4.803 ± 1.656
PPA + BIF	5.860 ± 0.697*abc	4.730 ± 0.395
PPA + Lacto	6.449 ± 0.818ab	4.706 ± 0.398
PPA + Mix	5.451 ± 0.481*abc	4.671 ± 0.696

(a) Control vs all groups, (b) PPA vs all groups, and (c) difference between all studied groups.

*The mean difference is significant at *p* <0.001.

**Figure 1:**
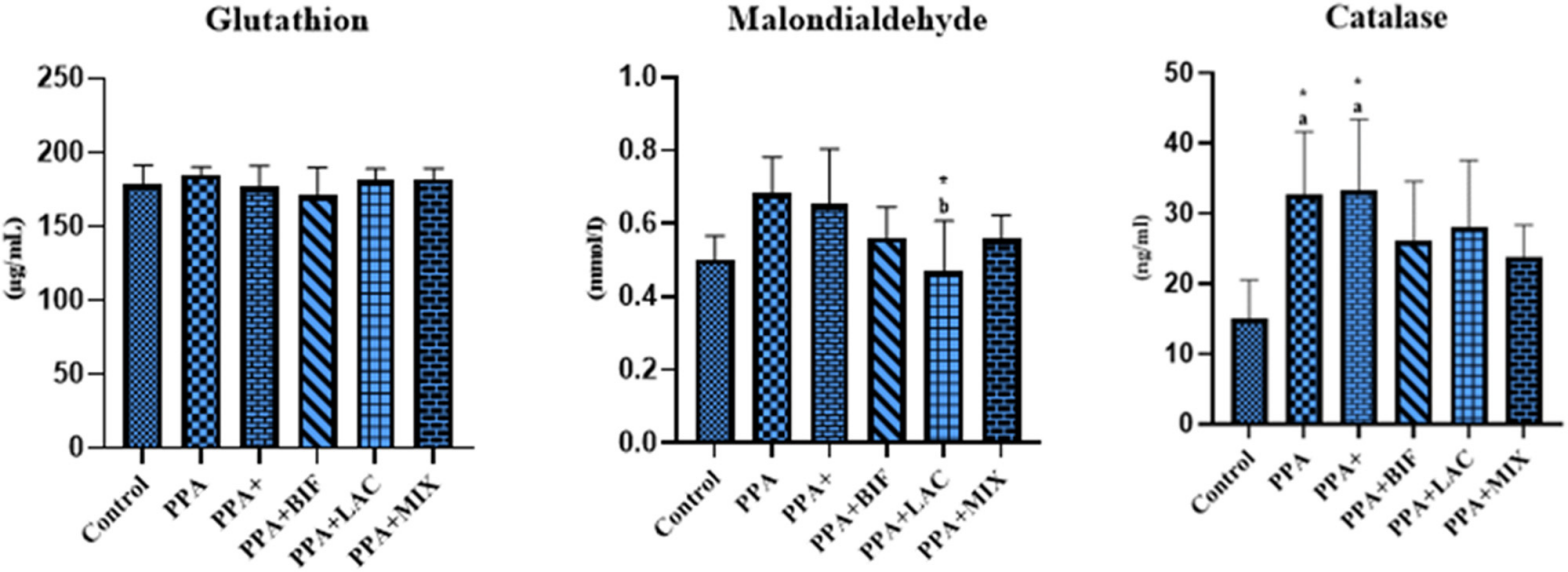
Impact of probiotics in ameliorating GSH, MDA, and catalase in PPA-induced rodent of ASD: (a) means significantly different compared to controls and (b) significantly different compared to the PPA group.

Figure 1 shows that, compared to the PPA-treated group, the *Lactobacillus*-treated group had much reduced MDA, a marker of oxidative stress; however, there was no discernible difference when compared to the control group.

**Figure 2:**
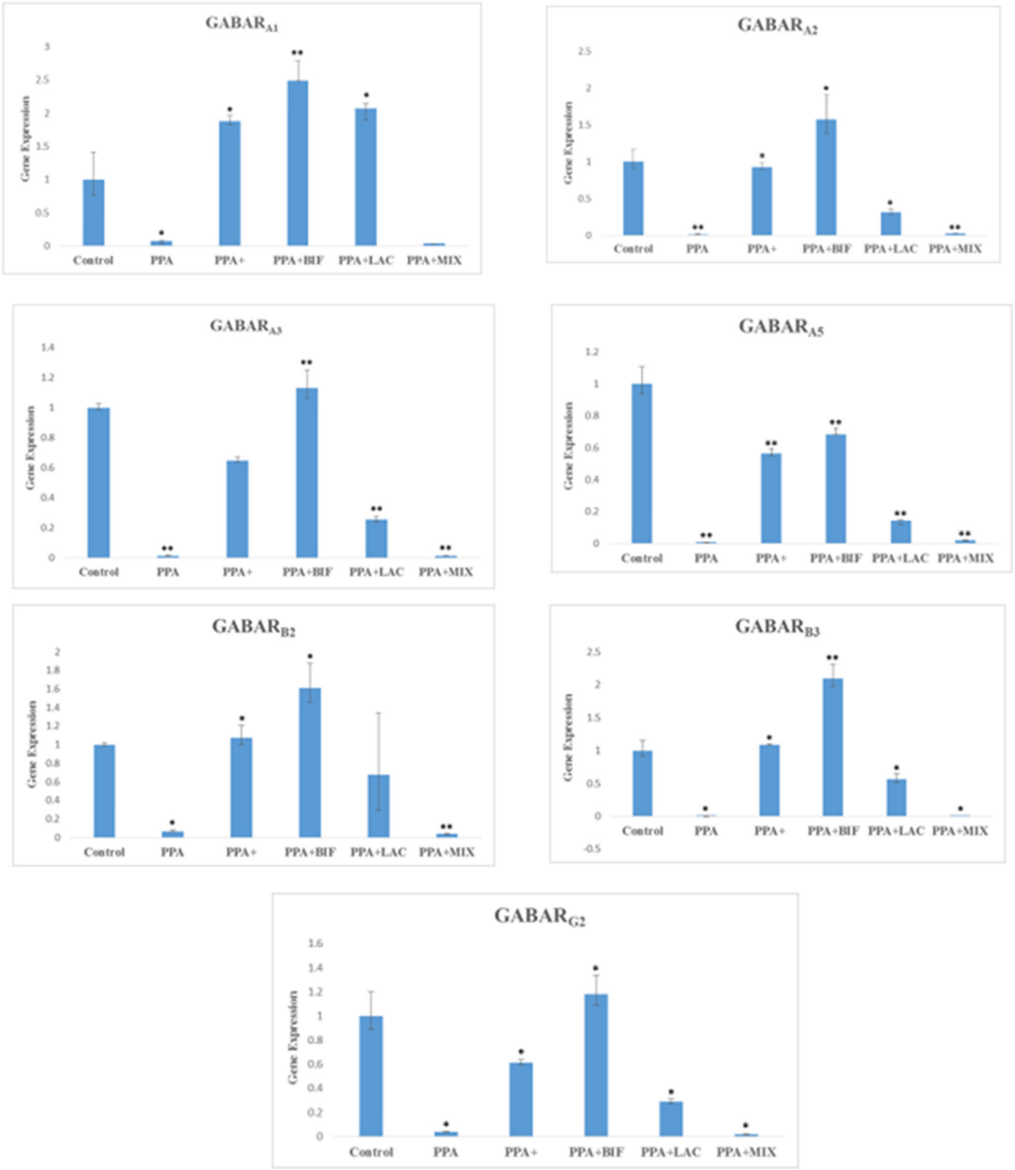
Impact of probiotic therapy on the chosen subunits of GABARG, GABARB, and GABARA gene expression in male western albino juvenile rats’ brain homogenates across all groups. At *p* < 0.001, the mean difference is significant (*), and at *p̂* 0.0001, it is significant (**). (Represented with permission from: Bin-Khattaf et al. [28]).

Figure 2 demonstrates the significant increase in GABA and GABA receptors in probiotic-treated rats, with *Bifidobacterium* strains being the most effective, followed by the probiotic mixture, while *Lactobacillus* was the least effective.

With zonulin, occludin, and GSH as the dependent variables, respectively, multiple regression analysis using the stepwise approach was carried out (Tables 2–4).

**Table 2:** Multiple regression using stepwise method for zonulin as a dependent variable

Predictor variable	Coefficient	S.E.	*P* value	Adjusted *R* ^2^	95% CI	
					Lower	Upper
GABARA1	2.026	0.586	0.002	0.244	0.834	3.218
GABARA1	3.170	0.600	0.000	0.438	1.947	4.393
GABARA5	−5.603	1.591	0.001		−8.843	−2.362
GABARA1	1.877	0.786	0.023	0.508	0.273	3.481
GABARA5	−14.489	4.064	0.001		−22.777	−6.201
GABARA3	9.365	3.984	0.025		1.238	17.491
GABARA1	3.170	0.894	0.001	0.578	1.344	4.995
GABARA5	−15.322	3.775	0.000		−23.032	−7.612
GABARA3	12.592	3.908	0.003		4.610	20.573
GABARA2	−4.053	1.627	0.019		−7.377	−0.730

**Table 3:** Multiple regression using stepwise method for occludin as a dependent variable

Predictor variable	Coefficient	S.E.	*P* value	Adjusted *R* ^2^	95% CI	
					Lower	Upper
GABARA1	1.020	0.381	0.011	0.154	0.245	1.794
GABARA1	1.761	0.390	0.000	0.370	0.966	2.556
GABARA5	−3.634	1.034	0.001		−5.741	−1.528

**Table 4:** Multiple regression using stepwise method for GSH as a dependent variable

Predictor variable	Coefficient	S.E.	*P* value	Adjusted *R* ^2^	95% CI	
					Lower	Upper
GABARA2	−7.762	3.142	0.019	0.131	−14.154	−1.370

All of the examined GABA receptors were responsible for the considerable change in zonulin, a measure of enhanced intestinal permeability, as mentioned in Table 2. The adjusted *R* ^2^ value of 0.578 in Table 2 indicates that GABA receptors as predictor variables significantly account for 57.8% of the change in zonulin.

Similar to this, but to a lesser degree, as mentioned in Table 3, adjusted *R* ^2^ value of 0.370 indicates that GABA1 and GABA5 each account for 37% of the change in occludin.

## 4 Discussion

Over the past few years, data have emerged from the literature suggesting a connection between dysbiosis, GI tract disorders, and an increased risk of diseases affecting the central nervous system. Obviously, a growing body of research has suggested that zonulin may have a significant role in the etiology of a number of microbial–gut–brain axis illnesses.

The effects of *Bifidobacteria* and *Lactobacillus*, either separately or in combination, on serum zonulin and occludin, biomarkers of intestinal permeability, as caused by experimentally administered neurotoxic doses of PPA, are displayed in Table 1. Although the PPA-induced ASD rodent model had substantially larger levels of both variables, it is still easy to detect that both probiotics had anti-gut leakiness effects, as seen by significantly lower blood zonulin levels. The findings of Fattorusso et al. [30] revealed that ASD patients’ plasma had greater zonulin concentrations and provide credence to the idea that zonulin levels are correlated with the severity of ASD symptoms [30] The reported anti-gut leakiness effects in *Bifidobacteria*-treated group could find support in multiple studies, which proved that exogenous *Bifidobacteria* supplementation enhances the function of the intestinal barrier and reduces the transfer of bacteria and endotoxins in rats injured by heat [31]. In a rat model of necrotizing enterocolitis, *B. bifidum* enhances intestinal integrity [32].

The reported anti-gut leakiness effects in the *Lactobacillus*-treated group in the current study can be supported by the fact that LGG is one of the best-studied probiotic bacteria in clinical trials for treating and/or preventing several intestinal disorders, including inflammatory bowel disease and diarrhea [33]. Figure 1 demonstrates that, in comparison with the PPA-treated group, the *Lactobacillus*-treated group had much less MDA, a measure of oxidative stress; nevertheless, there was no noticeable difference when compared to the control group. This is in accordance with Seth et al. [34], which proved that LGG stops oxidative stress-induced damage to barrier function and TJs in Caco-2 cell monolayers.

While GSH did not demonstrate any significant changes among the six studied groups, catalase was significantly higher in PPA-treated groups compared to the control healthy group. Both *Bifidobacteria* and *Lactobacillus* probiotics were effective in normalizing the activity of catalase as antioxidant enzyme. The unexpected increase of catalase in response to PPA-induced neurotoxicity could be attributed to the phenomenon that oxidative stress may be able to trigger catalase activity. This is further reinforced by the fact that free radicals can either inhibit enzyme function or produce an excess of enzymes in attempt to remove too many oxidative molecules. Both signaling pathways are connected with high MDA levels and indicate oxidative stress. Dietary probiotics and prebiotics may also have an impact on oxidative stress indicators. Thus, the remarkable increase of catalase in PPA-treated groups could support PPA oxidative effect previously reported by El-Ansary et al. [24], and the much lower catalase in *Bifidobacteria* and *Lactobacillus*-treated groups could be related to their antioxidant effects recently reported by Alsubaiei et al. [35].

Through the activation of GABA receptors, GABA can regulate immune cell function, inflammation, and GI motility and permeability. This may have an inhibitory or stimulatory effect on neuronal activity, and this could open the door to the development of treatment approaches that specifically target the “neuroimmune dialogue” in the gut [36]. Based on this, it was interesting to relate the recorded serum zonulin and occludin levels (Table 1), as markers of increased intestinal permeability, to the gene expression of brain GABA receptors previously reported in our recent published work (Figure 2), in an attempt to highlight the relationship between gut leakiness, the brain GABAergic signaling system, and a crucial bacterium found to have regulatory effects on this system through the gut–brain axis [25]. When compared to a PPA-induced animal model, treatment with *Bifidobacteria* and *Lactobacillus* was efficient in reducing gut leakiness, lowering serum levels of zonulin and occludin while concurrently exhibiting much higher gene expression of brain GABA receptors. This proposed relationship can find great support through considering the work of Bravo et al. [37], which reported that oral administration of LGG altered the brain mRNA expression of GABA_A_ and GABA_B_ receptors while decreasing depressive and anxiety-related behaviors in mice through the vagus nerve. This could help to suggest new therapeutic options for the favorable effects of probiotics on newborn neurodevelopmental processes against LPS-induced inflammatory responses and altered gut microbiota in the prenatal period. This suggestion is in good agreement with recent research that supports the use of probiotics to treat neuroinflammation in brain tissue by lowering levels of Aβ1-42, amyloid-beta precursor protein, γ secretase, and β-secretase, as well as fecal calprotectin, a biomarker of intestinal flora disruption [38].

Moreover, *Bifidobacteria* produces GABA through the enzymatic decarboxylation of glutamate, and daily oral administration of *Bifidobacterium* strain modulated sensory neuron activity, unbalanced GABA/glutamate, and glutamate excitotoxicity as a neurochemical feature of PPA neurotoxicity [39,40].

In an attempt to support this suggested relationship, multiple regression analyses using stepwise method were performed with zonulin, occludin, and GSH as dependent variables, respectively (Tables 2–4).

Table 2 shows that the significant change in zonulin, a marker of increased intestinal permeability, was caused by all of the tested GABA receptors. GABA receptors as predictor variables significantly account for 57.8% of the change in zonulin, as shown by the adjusted *R* ^2^ value of 0.578, as mentioned in Table 2.

Similarly, but to a lesser extent, GABA1 and GABA5 contribute to 37% of the change in occludin, as shown by the adjusted *R* ^2^ value of 0.370 in Table 3.

This is consistent with a study that found that behavioral abnormalities in male Shank3-knockout mice, a rodent model of ASD, were controlled after the injection of *Lactobacillus reuteri*, a probiotic that may adjust the significant changes in GABA receptor gene expression [41]. Furthermore, dysbiosis in adult mice housed in ordinary conditions causes abnormal behaviors, alterations in intestinal barrier function, and activation of the brain’s resident immune cells, known as microglia [42].

Oxidizing and reducing chemicals, which are now involved in cell metabolism and signaling pathways, have the ability to modulate rapid inhibitory neurotransmission in the nervous system via GABA receptor. A number of *in vitro* studies have revealed that various redox chemicals, such as redox metabolites and reactive oxygen and nitrogen species, alter phasic and tonic responses mediated by neuronal GABAA receptors via both presynaptic and postsynaptic pathways [43]. In relation to this phenomenon, interestingly, Table 4 demonstrates the contribution of GABARA2 in 13.1% of the change of GSH as dependent variable in multiple regression analysis. Based on this, we can suggest that redox signaling is hypothesized to be a homeostatic process that modulates the function of synaptic and extrasynaptic GABAA receptors in both normal and pathological situations. Our study is reinforced by research demonstrating that enhanced barrier permeability in animal models of autism can transfer LPS and bacterial toxins mostly produced by *Proteobacteria* and *Clostridium difficile* to brain tissues, activating the TLR 4/MyD88/NF-κB pathway and establishing a pro-inflammatory milieu, leading to neurodegeneration as a mechanism through which systemic enteric inflammation might cause nerve dysfunction [44,45]. Miranda-Ribera et al. [46] proved that the correction of gut microbiota can partially alleviate behavioral problems, altered BBB integrity, and dysbiosis, which are all linked to zonulin-dependent changes in gut permeability. They propose that the zonulin model may be utilized to study how the brain and microbiome/gut interact with neurobehavioral and neuroinflammatory illnesses.

## 5 Conclusions

The current investigation addresses the possible uses of supplements including pure *Lactobacillus bulgaricus*, a probiotic combination called ProtexinR, and *Bifidobacterium infantis* in treating neurophenotypes associated with PPA neurotoxicity. It accomplishes this by highlighting the supplements’ capacity to reduce elevated intestinal permeability, enhance GABA receptor expression, and lessen oxidative stress.
